# Silicon and Salinity: Crosstalk in Crop-Mediated Stress Tolerance Mechanisms

**DOI:** 10.3389/fpls.2019.01429

**Published:** 2019-11-07

**Authors:** Adil Khan, Abdul Latif Khan, Sowbiya Muneer, Yoon-Ha Kim, Ahmed Al-Rawahi, Ahmed Al-Harrasi

**Affiliations:** ^1^Natural & Medical Sciences Research Center, University of Nizwa, Nizwa, Oman; ^2^School of Agricultural Innovations and Advanced Learning, Vellore Institute of Technology, Vellore, India; ^3^School of Applied Biosciences, Kyungpook National University, Daegu, South Korea

**Keywords:** silicon, salinity, stress tolerance, antioxidant, reactive oxygen species

## Abstract

Salinity stress hinders the growth potential and productivity of crop plants by influencing photosynthesis, disturbing the osmotic and ionic concentrations, producing excessive oxidants and radicals, regulating endogenous phytohormonal functions, counteracting essential metabolic pathways, and manipulating the patterns of gene expression. In response, plants adopt counter mechanistic cascades of physio-biochemical and molecular signaling to overcome salinity stress; however, continued exposure can overwhelm the defense system, resulting in cell death and the collapse of essential apparatuses. Improving plant vigor and defense responses can thus increase plant stress tolerance and productivity. Alternatively, the quasi-essential element silicon (Si)—the second-most abundant element in the Earth’s crust—is utilized by plants and applied exogenously to combat salinity stress and improve plant growth by enhancing physiological, metabolomic, and molecular responses. In the present review, we elucidate the potential role of Si in ameliorating salinity stress in crops and the possible mechanisms underlying Si-associated stress tolerance in plants. This review also underlines the need for future research to evaluate the role of Si in salinity stress in plants and the identification of gaps in the understanding of this process as a whole at a broader field level.

## Introduction

Soil salinity is one of the major abiotic stress that hinders crop growth and productivity worldwide (Ahmad et al., 2019b). It has been reported that approximately 20% of irrigated land is salt-affected, which represents one-third of food-producing land (Shrivastava and Kumar, 2015; Gregory et al., 2018). Further to this, half of all the fertile land will be affected by salinity by the middle of the 21^st^ century (Shahid et al., 2018). The salt-affected areas are increasing at a rate of 10% annually for various reasons, including low precipitation, the weathering of native rocks, high surface evaporation, poor cultural practices, and irrigation using saline water (Shrivastava and Kumar, 2015). This issue has been further aggravated by the continued trends in global warming and climatic changes. Thus, enhancing crop plant tolerance to abiotic stresses is an important challenge to overcome deteriorating food production system and to meet the demand of food supply for ever-increasing world population (Shah and Wu, 2019). To ensure sustainable food supply, a considerable 50% increase in the grain yields of major crop plants such as wheat, rice, and maize (Godfray et al., 2010; Shrivastava and Kumar, 2015) is required. However, soil, which represents a major ecosystem that often operates at a subsistence level in the growth of crops, is often compromised by salinity.

Salinity stress affects the morphological, physiological, and biochemical processes of plants (Singh and Chatrath, 2001; Ashraf, 2004). High salinity not only decreases plant growth, biomass, yield, photosynthesis, and water use efficiency, but also leads to physiological drought and ion toxicity in plants, thus reducing agricultural productivity and yields (Shahid et al., 2018). Salinity stress also causes ionic imbalances, the osmotic effect, water use insufficiency, and nutrient (e.g. N, Ca, K, P, Fe, and Zn) deficiency, which ultimately leads to oxidative stress in plants (Rehman et al., 2019). Reactive oxygen species (ROS) are produced in plant cells under normal physiological conditions, either in a radical or non-radical form (Winterbourn, 2019). However, excessive ROS production leads to oxidative damage to the proteins, lipids, nucleic acids, and plasma membrane of the cell. During normal cellular metabolism, the plant produces several antioxidant enzymes such as superoxide dismutase (SOD), catalase (CAT), peroxidase (POD), glutathione peroxidase (GPX), glutathione reductase (GR), and ascorbate peroxidase (APX) for the detoxification of ROS. In addition to high ROS production, salinity stress significantly reduces the uptake of phosphorus (P) and potassium (K), while increasing the uptake of toxic elements such as sodium (Na^+^) and chlorine (Cl^-^), which have negative effects on plant growth and productivity. High concentrations of Na^+^ create osmotic stress, which consequently leads to cell death (Munns, 2002; Ahanger et al., 2017). Photosynthesis machinery is also affected by salinity stress, mainly due to the reduction in the leaf area, stomatal conductance, and chlorophyll levels, and to a lesser extent by the decrease in photosystem II efficiency (Netondo et al., 2004). Any mechanisms that maintain optimal K^+^/Na^+^ ratios, nutrient concentrations, and ROS production in plants are thus likely to provide effective resistance against salinity stress (Assaha et al., 2017).

Various mitigation and adaptation approaches have been used to overcome these negative impacts of high soil salinity (Wang et al., 2019). The use of different approaches to alleviate the negative effects of salinity is likely to ensure the sustainable production of food, but salinity stress management is very challenging due to its multigenic and quantitative nature (Ahmad and Rasool, 2014). Strategies have been reported for the amelioration of the negative effects of salinity on plants, such as developing salt-tolerant crops, transgenic varieties, plant growth-promoting bacteria, endophytes, the leaching of salt from the root zone, and micro-jet irrigation to optimize the use of water (Chanchal Malhotra et al., 2016; Ibrahim et al., 2016). However, very little knowledge still exist about the mineral status and dynamics of plants and their salinity tolerance (Manchanda and Garg, 2008).

The exogenous application of silicon (Si) has been a recent eco-friendly approach to enhance the salinity stress response in plants (Almeida et al., 2017). Silicon is the second-most abundant element on Earth, making up 27.7% of the Earth’s crust, second only to oxygen. It occurs naturally in the form of complex silicate minerals, either in crystalline, amorphous, or poorly crystalline phases (Sommer et al., 2006; Frew et al., 2018). Most soils contain an Si concentration ranging from 14 to 20 mg Si/L (Montpetit et al., 2012; Vivancos et al., 2015). Silicon is available in the form of silicic acid (Si(OH)_4_) in soil solutions in a concentration range of 0.1–0.6 mM (Luyckx et al., 2017). Plant roots absorb Si in the form of monosilicic acid *via* aquaporin-type channels (NOD26-like intrinsic proteins, NIPs; Deshmukh et al., 2013). However, the translocation and movement of Si is a very slow process, thus amendment with exogenous soluble Si is needed in order to ameliorate stress conditions and improve the yields of crops. In the early 1900s, Si was recognized as one of the 15 most important elements needed for plant life (Debona et al., 2017). All plants grown in soil contain a certain level of Si in their tissues, as reported for more than 44 angiosperm clades that represent over 100 orders or families (Debona et al., 2017). Many terrestrial plants accumulate a noticeable concentration of Si (Etesami and Jeong, 2018) while, in most dicots, less than 0.1% Si is found based on dry weight. A large number of grass species have the capacity to accumulate up to 10% of Si (Vivancos et al., 2015), with rice the most effective Si accumulator (Kaur and Greger, 2019). Si is coming to the fore as a true “Cinderella” element (Artyszak, 2018), gaining interest among scientists from across the world because of its effective role in plant physiology, nutrition, and defense response. The valuable role of exogenous Si on plant growth and yield has been well-documented in the literature, but its true potential lies in the amelioration of abiotic and biotic stresses (Rodrigues and Datnoff, 2015; Khan et al., 2018; Wu et al., 2019).

The available literature has clearly described the role of Si in combating abiotic and biotic stresses in plants; however, it has still not been listed as an essential element for plants because no clear evidence has been presented, unlike other essential elements. Numerous studies have reported that Si increases plant resistance against biotic and biotic stresses (Epstein, 1999; Ma and Takahashi, 2002; Ma and Yamaji, 2006), such as salt and drought (Zhu and Gong, 2014; Rizwan et al., 2015), extreme temperature stress (Ma, 2004), nutrient deficiency (Marafon and Endres, 2013), aluminum toxicity (Galvez and Clark, 1991; Shen et al., 2014; Pontigo et al., 2015; Pontigo et al., 2017), disease resistance (Van Bockhaven et al., 2012; Marafon and Endres, 2013), and resistance to damage by wild rabbits (Cotterill et al., 2007). It also contributes to plant growth in different ways by enhancing multiple adaptive responses, such as antioxidant activity, mineral uptake, organic acid anion and phenolic compound exudation, the photosynthesis rate, the accumulation of compatible solutes, water status, and hormonal regulation (Barcelo et al., 1993; Cocker et al., 1998; Kidd et al., 2001; Al-Aghabary et al., 2005; Shahnaz et al., 2011; Shen et al., 2014; Sivanesan and Jeong, 2014; Kim et al., 2016; Kim et al., 2017; Tripathi et al., 2017; Ahanger et al., 2018; Ahmad et al., 2019a) and significantly reducing the adverse effects of salinity on chlorophyll levels and plant biomass (Seal et al., 2018). Despite this, most of these findings are scattered and need to come up with a comprehensive image of progress made on this topic. There have been some recent review articles published on Si, such as those by Etesami and Jeong (2018) and Malhotra and Kapoor (2019), but they do not address the crosstalk of physio-molecular functions in response to salinity stress. In this review, we focus on studies that have investigated plant metabolism and physiology under salinity stress and have elucidated the complex mechanisms and interactions involving Si in the amelioration of the detrimental effect of salinity on crops.

## Plant Physiology Under Salinity Stress

**Figure 1:**
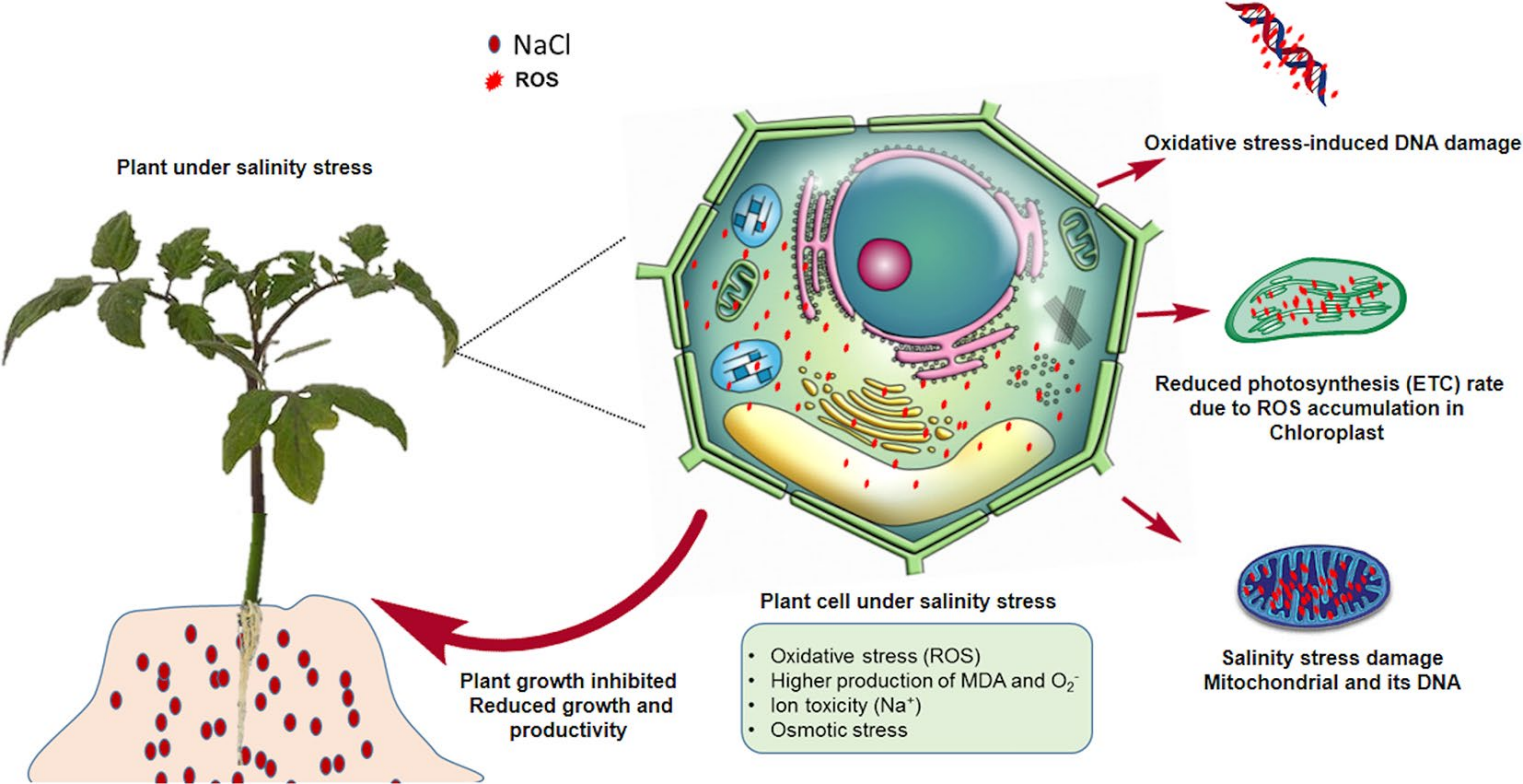
Higher ROS levels in plants under salinity stress conditions. Toxic levels of ROS stunt plant growth by inhibiting the electron transport chain, and photosynthesis in plastids, and by causing mutations in DNA and damaging mitochondria.

High salinity can increase the uptake of Na^+^ and Cl^-^ from the soil, consequently suppressing the transport of other essential nutrients such as N, P, K, and Ca (Shrivastava and Kumar, 2015; Safdar et al., 2019). The resulting ionic and secondary stresses, such as nutritional imbalances, disturb the overall osmotic balance, resulting in physiological drought, i.e. the prevention of water uptake (Riaz et al., 2019). In the case of halophytic plants that are resistant to sodium toxicity, osmotic stress is a possible reason for the inhibition of their growth. Photosynthesis is also affected by salinity because of the reduction in chlorophyll content, stomatal conductance, and leaf area. Photosystem II is also primarily affected by salinity (Najar et al., 2019). Salinity affects reproductive development by inhibiting microsporogenesis, elongating stamen filaments, accelerating programmed cell death, and promoting the senescence of fertilized embryos and ovule abortion (Suo et al., 2017). Under saline conditions, the absorption of atmospheric carbon dioxide is reduced, leading to greater stomatal closure and the lower utilization of NADPH *via* the Calvin cycle (Suo et al., 2017). These conditions favor the electron acceptor behavior of molecular oxygen, leading to the accumulation of ROS. High ROS levels can damage essential macromolecules necessary for the normal growth of plants by altering their metabolism *via* oxidative lipids, nucleic acids, and protein damage (**Figure 1**). ROS are produced continuously during normal metabolic events in peroxisomes, mitochondria, and the cytoplasm (Saini et al., 2018). Other processes that are affected by salt stress include stem and root growth, ion transport, plant morphology, the enzymatic activity of solutes, cell structure maturation, and nutrient uptake. A significant reduction in stem height and root length has also been observed for cases of high osmotic stress (Shrivastava and Kumar, 2015). In response to higher concentrations of NaCl in the soil, sodium uptake by the roots is enhanced while phosphorus, nitrogen, magnesium, and potassium uptake is lowered significantly, leading to the disruption of the intracellular ionic balance (Jayakannan et al., 2013). Under these circumstances, plant roots cannot absorb enough water and significant energy is required to adjust the osmotic balance *via* compatible solute accumulation (Acosta-Motos et al., 2017).

## Plant Cellular Mechanisms That Improve Tolerance to Salinity Stress

The salt tolerance level varies from species to species and even different cultivars, whereas the individual plants of the same cultivar would show a variation. Some species such as redbay, *Anemopsis californica*, and *Quercus geminate* are more resistant to high levels of salt (Roy et al., 2014), while others, such as *Schoenus* spp., *Polypogon viridis*, and *Juncus* spp. are sensitive or even hypersensitive to low salinity levels (Gibson et al., 1984). The biochemical and physiological mechanisms underlying plant salinity tolerance can be divided into those that minimize osmotic stress and ion imbalances and those that act on secondary effects caused by this stress, such as imbalances in plant nutrition and oxidative stress. The principle mechanisms are ion homeostasis, ion uptake and transport, the biosynthesis of compatible solutes and osmo-protectants, antioxidant enzyme activation, antioxidant and polyamine synthesis, nitric oxide (NO) generation, and hormonal alterations. Past research that has elucidated these mechanisms is briefly summarized below.

### Significance of Ion Homeostasis in Plant Tolerance to Salinity

Under salinity stress, plants accumulate high levels of Na^+^ and Cl^-^ compared to other cations like K^+^ and Ca^2+^ (Tavakkoli et al., 2010), creating physiological problems and ion imbalances (James et al., 2011; Hasegawa, 2013; Ahmad et al., 2018). Neither halophytes nor glycophytes can tolerate high salt concentrations in the cytoplasm. H^+^ pumps (Na^+^/H^+^ antiporters) are responsible for the transport of Na^+^ ions from the cytoplasm to the vacuoles. The vacuolar membrane has two types of H^+^ pump, i.e. *vacuolar-type H* *^+^* *-ATPase* (*V-ATPase*) and *vacuolar pyrophosphatase* (*V-PPase*; Graus et al., 2018). *V-ATPase* pumps occur in high numbers in plant cells. H^+^ pumps are responsible not only for maintaining solute homeostasis under normal conditions but also for facilitating secondary transport and vesicle fusion. However, during stress, the survival of plants is determined by the action of *V-ATPase* pumps (Bozza et al., 2019). Otoch et al. (2001) reported that *V-ATPase* pump activity increased in *Vigna unguiculata* seedlings (a hypocotyl) when exposed to salinity, while *V-PPase* pump activity was inhibited under similar conditions. In *Suaeda salsa* (a halophyte), the activity of *V-ATPase* pumps was upregulated while that of *V-PPase* pumps were downregulated (Wang et al., 2001a). Hence, both H^+^ pumps are responsible for maintaining solute homeostasis at the cellular level (Graus et al., 2018).

**Figure 2:**
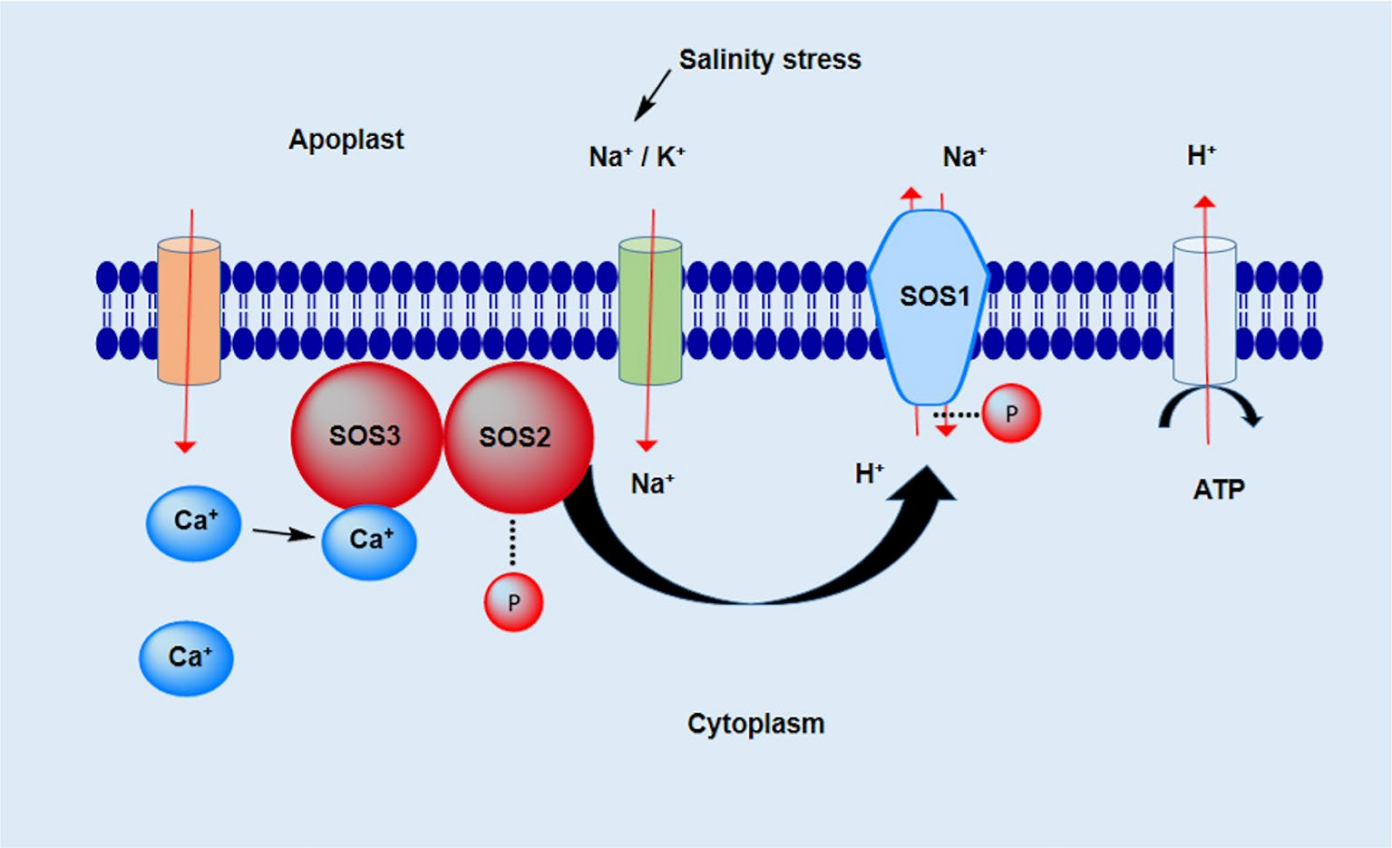
SOS pathway under salinity stress. The transport of ions across the membrane is conducted by various carrier proteins, e.g. channel proteins, antiporters, and symporters. The ion hemostasis (Na^+^, K^+^, and Ca^2+^) in the cell is crucial for its survival under salt-stress conditions.

Several studies have reported that salt plays a role in the *Salt Overly Sensitive* (*SOS*) pathway, which consists of the three proteins *SOS1*, *SOS2*, and *SOS3*. The cytoplasmic membrane Na^+^/H^+^ antiporter encodes *SOS1*, which plays an important role in the regulation of Na^+^ efflux at the cellular level (Numan et al., 2018). However, *SOS1* is also essential for the regulation of the long-distance diffusion of Na^+^ between the roots and shoots (Abbas et al., 2017). In saline conditions, the overexpression of *SOS1* increase salt tolerance levels (Fan et al., 2019). The salinity stress activates the Ca^2+^ signaling pathway, which consequently stimulates the production of threonine/serine kinase encoded by the *SOS2* gene, which consists of a regulatory domain on the C-terminal and a catalytic domain on the N-terminal (Zhu, 2016). The third gene *SOS3* encodes a myristoylated Ca^2+^ binding protein that contains a myristoylation site on the N-terminus. This site is very important for the conferral of salinity resistance on plants (Ishitani et al., 2000). The *SOS2* protein consists of a FISL motif on the C-terminal, composed of 21 amino acids, and provides an interaction site for the binding of Ca^2+^ to the *SOS3* protein (Köster et al., 2019). The interaction between the *SOS2* and *SOS3* proteins triggers protein kinase activation. This activated protein kinase is responsible for the phosphorylation of *SOS1* protein and eventually leads to an increase in the efflux of Na^+^ and a decrease in Na^+^ ion toxicity (**Figure 2**).

The role of *SOS1* in controlling ion homeostasis has been demonstrated through a combination of biochemical, genetic, and physiological analyses. Using yeast mutant strains and isolated plasma-membrane vesicles, *SOS1* was first shown to be able to specifically transport Na^+^ out of cells under salt stress (Qiu et al., 2002; Shi et al., 2002). These proteins not only regulate ion hemostasis but are also essential for the regulation of pH homeostasis, vacuole functions, and membrane vesicle trafficking (Oh et al., 2010). In addition to the SOS stress signaling pathway, another method of developing resistance to salinity stress has been reported for many plants that maintain a minimum ion concentration in the cytosol. During stress conditions, membranes and their linked components maintain the ion concentration in the cytoplasm by regulating ion transport across the membrane (Sairam and Tyagi, 2004).

### Role of Antioxidants Under Salinity Stress

Plant salt stress can negatively affect the electron transport chain (ETC) in mitochondria and chloroplasts by unbalancing or completely distorting the regulation process (Numan et al., 2018). Molecular oxygen acts as an electron acceptor, which can lead to the overproduction of ROS, including hydroxyl radicals, singlet oxygen, superoxide radicals, and H_2_O_2_. ROS are powerful oxidizing compounds that can damage the plasma membrane and endomembrane systems (Ahanger and Agarwal, 2017; Foyer, 2018). However, they also act as signals of stress, thus activating the antioxidant enzymes SOD, GPX, APX, CAT, and GR and non-enzymatic compounds (e.g. glutathione, non-protein amino acids, ascorbic acid, and phenolic compounds) to sustain a balanced level of ROS in cells under both normal and stress conditions (Caverzan et al., 2016; Ahanger et al., 2018). These enzymes are responsible for removing ROS that have accumulated in plants due to salinity stress. Tuteja et al. (2013) reported that the proteins DESD-box helicase and *OsSUV3* dual helicase increase the tolerance to salinity by maintaining or improving photosynthesis and antioxidant enzyme machinery. Thus, understanding the mechanisms that regulate ROS signaling at the cellular level during stress can provide a more powerful approach for developing resistance to high salt levels.

### Role of Nitric Oxide (NO) in Salt Tolerance in Plants

Nitric oxide (NO) is small volatile gaseous molecule that is essential for the maintenance of various physiological and biochemical mechanisms at the cellular level in plants, e.g. root growth, stomata closure, respiration, stress signaling, ﬂowering, cell death, seed germination, and stress responses (Besson-Bard et al., 2008; Zhao et al., 2009). Under stress conditions, NO either directly or indirectly regulates many genes involved in developing tolerance to salinity stress, including various redox-related and antioxidant enzyme genes (e.g. GPX, GR, SOD, CAT, and APX), and suppresses lipid peroxidation or malondialdehyde (MDA), consequently restoring normal plant growth (Bajguz, 2014). NO increases plasma membrane expression and/or tonoplast *H* *^+^* *-ATPase* and *H* *^+^* *-PPase* to maintain a high K^+^/Na^+^ ratio in the cytoplasm in response to salinity (Sung and Hong, 2010; Zhang et al., 2017). NO also assists the cell in accumulating various compatible solutes, such as proline, organic osmolytes, and soluble sugars, to facilitate cell turgor and balanced water acquisition (Guo et al., 2005).

### Improved Salinity Tolerance *via* the Accumulation of Compatible Solutes

Salinity stress promotes the accumulation of compatible solutes or osmolytes, a set of chemically altered organic compounds that are polar or uncharged in nature that do not affect the biochemical processes of the cell at high concentrations (Ashraf et al., 2011; Vyrides and Stuckey, 2017). They generally include mannitol, proline (Hoque et al., 2007; Nounjan et al., 2012; Tahir et al., 2012), glycine betaine (Khan et al., 2000), sugar (Ford, 1984; Wang and Nii, 2000), raffinose oligosaccharides, and N-containing compounds such as amino acids, polyamines, and polyols (Saxena et al., 2013). Organic osmolytes are produced in variable amounts between diﬀerent plant species. For example, the quaternary ammonium compound beta-alanine is found only in a few species belonging to the *Plumbaginaceae* family (Hanson et al., 1994), whereas proline accumulation occurs in a diverse range of plants (Saxena et al., 2013). The concentration of compatible solutes is regulated either by irrevocable synthesis or by a process of degradation and synthesis. The rate at which compatible solutes accumulate in the cell is determined by the external osmolarity, and the current understanding of the mode of action of these solutes includes providing osmotic adjustment *via* continuous water inﬂux, stabilizing proteins and the cell structure, and scavenging ROS when under salt stress (Turkan, 2011).

Of the nitrogen-containing compounds, some amino acids accumulate to higher levels under salt stress. The concentration of arginine, cysteine, and methionine decrease under salt stress, whilst that of proline increases (El-Shintinawy and El-Shourbagy, 2001). These amino acids play a vital role in the salt stress response by instigating K^+^ homeostasis, leading to a plant’s adaptation to salinity by reducing NaCl-induced K^+^ efflux (Cuin and Shabala, 2007). Of these, proline has a substantial role, and its concentration rises significantly in many plants. Additionally, glycine betaine is another primary osmoprotectant, synthesized in response to salinity stress by many plants. It maintains the osmotic cell status to improve the response to abiotic stress (Kumar et al., 2018). For example, Rahman et al. (2002) reported the positive eﬀect of glycine betaine on the ultrastructure of *Oryza sativa* seedlings when exposed to salt stress.

### Phytohormone Regulation Under Salinity Stress

Phytohormones play an important role in plant growth and development under both normal and stressful conditions. In the literature, they are often regarded as plant growth regulators (compounds that derive from plant biosynthetic pathways; Peleg and Blumwald, 2011). Several hormones such as abscisic acid (ABA), indole acetic acid (IAA), salicylic acid (SA), brassinosteroids (BR), cytokinins (CKs), ethylene (ETHY), gibberellic acid (GA), and jasmonic acid (JA; Iqbal et al., 2014) have been reported to regulate plant growth and development in a coordinated fashion by either acting locally or being transported to another site within the plant (Fahad et al., 2015). Harsh conditions disrupt the production and distribution of hormones that may promote specific protective mechanisms in plants (Eyidogan et al., 2012; Fahad et al., 2015). Thus, plant stress-related hormones have an important role in mediating plant responses to abiotic stress, by which plants attempt to avoid or survive stressful conditions and in doing so exhibit reduced growth so that the plant can focus its resources on withstanding the stress.

ABA acts as a cellular signaling or stress hormone, and exogenous application has been suggested for increasing salt tolerance (Sah et al., 2016). Endogenous ABA accumulates in various plants, especially in osmotic and salt stress conditions (Sah et al., 2016). ABA improves tolerance, partly due to the accumulation of ions and compatible solutes (such as proline and sugar) in the vacuoles of the root, neutralizing the uptake of Na^+^ and Cl^-^ (Chen et al., 2001; Gurmani et al., 2011; Cho et al., 2018). Similarly, the increased production of ABA can ameliorate the negative eﬀect of stress on photosynthesis. Fricke et al. (2004) reported that the increase in the ability of xylem to uptake water under saline conditions is hindered by ABA homeostasis. However, the role of ABA in the regulation of important cellular signals has been clearly demonstrated, controlling the expression of many important water and salt deficit responsive genes such as *cinnamyl alcohol dehydrogenase*, *9-cisepoxycarotenoid dioxygenase*, *zeaxanthin oxidase*, *molybdenum cofactor sulfurase*, and *ABA-aldehyde oxidase* through a calcium-dependent phosphorylation pathway (Dubos and Plomion, 2003; Ryu and Cho, 2015).

Another important hormone is GA; GA_3_ regulates ion uptake and the homeostasis of hormones under saline conditions in *Lycopersicon esculentum* and *T. aestivum* (Maggio et al., 2010). Similarly, Maggio et al. (2010) reported that GA_3_ applied to tomato plants reduced stomatal resistance and enhanced the water status. Iqbal and Ashraf (2013) reported that GA_3_ treatment under saline conditions modulated ion uptake and partitioning and hormone homeostasis in wheat. It has also been reported that plant phytohormones such as indole IAA respond to salinity stress in crop plants (Fahad et al., 2015). Additionally, IAA levels in the root system fall significantly after NaCl treatment in *Triticum aestivum* (Sakhabutdinova et al., 2003), *Oryzasativa* (Dunlap and Binzel, 1996), and *Esculentum* L. (Smirnoff, 1997). It has been observed that the activity of JA increased under salinity stress (Farhangi-Abriz and Ghassemi-Golezani, 2018). For example, JA treatment recovered the negative effect of salt on seedling development and photosynthetic activity in several cultivar crops (Yoon et al., 2009; Javid et al., 2011). These results are strongly indicative of the positive role of JA in salt stress responses in plants.

Other phytohormones, such as BR and SA, also play an important role in plant abiotic stress responses (Wani et al., 2016). SA controls different features of plant responses to stress *via* widespread signaling with other growth hormones (Horváth et al., 2007; Jayakannan et al., 2013). Undesirable salinity eﬀects may be alleviated by BR (Ashraf et al., 2010b; El-Mashad and Mohamed, 2012). The application of BR enhances the antioxidant activity of SOD, POX, APX, and GPX and the accumulation of non-enzymatic antioxidant compounds (tocopherol, ascorbate, and reduced glutathione; El-Mashad and Mohamed, 2012; Iqbal et al., 2014; Simura et al., 2018) (El-Mashad and Mohamed, 2012). Indeed, a stress signal triggers signal transduction cascades in plants, with phytohormones acting as baseline transducers (Fahad et al., 2015).

## Silicon Uptake, Transport, and Assimilation in Plants

The concentration of Si in soil is similar to that of macronutrients assimilated by plants (Sommer et al., 2006), but it cannot be directly absorbed (Mitani et al., 2005). Furthermore, Si absorption, accumulation, and transport capacity differ significantly between species (Ma and Takahashi, 2002). Generally, the concentration of monosilicic acid ranges from 0.1 to 0.6 mM in soil solutions in which Si is present as an uncharged monomeric molecule at pH 9. Many factors, such as temperature, pH, the presence of cations, water conditions, and the organic compounds present in solution directly influence solvable silicic acid formation in the soil and indirectly affect the accumulation rate in plants (Liu et al., 2003). The lower availability of Si in the soil was reported to be the probable reason for decreasing rice yields (Meena et al., 2014). Tropical and subtropical soils have low Si levels due to de-silication caused by weathering and leaching processes (Epstein, 1999), while an estimated 210–224 million tons of Si are taken out annually from the world’s arable soils (Meena et al., 2014).

All plants contain Si in considerable amounts in all parts such as the roots, shoots, and leaves but various levels are found in different species (Takahashi et al., 1990). The roots take up Si in silicic acid form and it is transported to the rest of the plant using active, passive, and rejective transport. Based on the take-up capacity of Si, plants are categorized as high, intermediate, or non-Si accumulators (Marafon and Endres, 2013; **Table 1**). Previously, the active uptake of Si has been demonstrated in different plants such as rice (Klotzbucher et al., 2018), wheat (Rains et al., 2006), maize (Mitani et al., 2009), and barley (Chiba et al., 2009), while tomato limits the transport of Si from the roots to shoots (Wang et al., 2015a). Liang et al. (2005a) and Mitani and Ma (2005) reported different results for cucumber plants. Liang et al. (2006) also reported both active and passive transport in rice, maize, sunflower, and wax gourd. These findings suggest that both the passive diffusion of silicic acid and transporter-mediated uptake are involved in the radial root transport of Si, with transporter-mediated Si uptake an energy-dependent process because metabolic inhibitors and low temperatures inhibit Si transport (Liang et al., 2006; Feng et al., 2011). Furthermore, these results suggest that the occurrence of both types of transporters is somehow dependent on the species and concentration of Si in the soil. Hence, the differences in Si uptake reported for cucumber plants might be due to the coexistence of both active and passive uptake in various cultivars or due to the concentration of Si in the soil.

**Table 1:** Plant categories based on Si uptake capacity (Bakhat et al., 2018).

> 1.5% Si High accumulator	1.5%–0.5% Intermediate accumulator	<1.5% of Si Non-accumulator
Rice	Pumpkins	Tomato
Ferns	Cucumber	Pansy
Horsetail	Rose	Begonia
Lentils	Squash	Grapes
Mosses	Chrysanthemums	Sunflower
Sugarcane	Soybean	Gerbera
Conifers	Zinnia	Petunia
Wheat	New Guinea Impatiens	Snapdragon
Spinach	Marigold	Geranium

**Figure 3:**
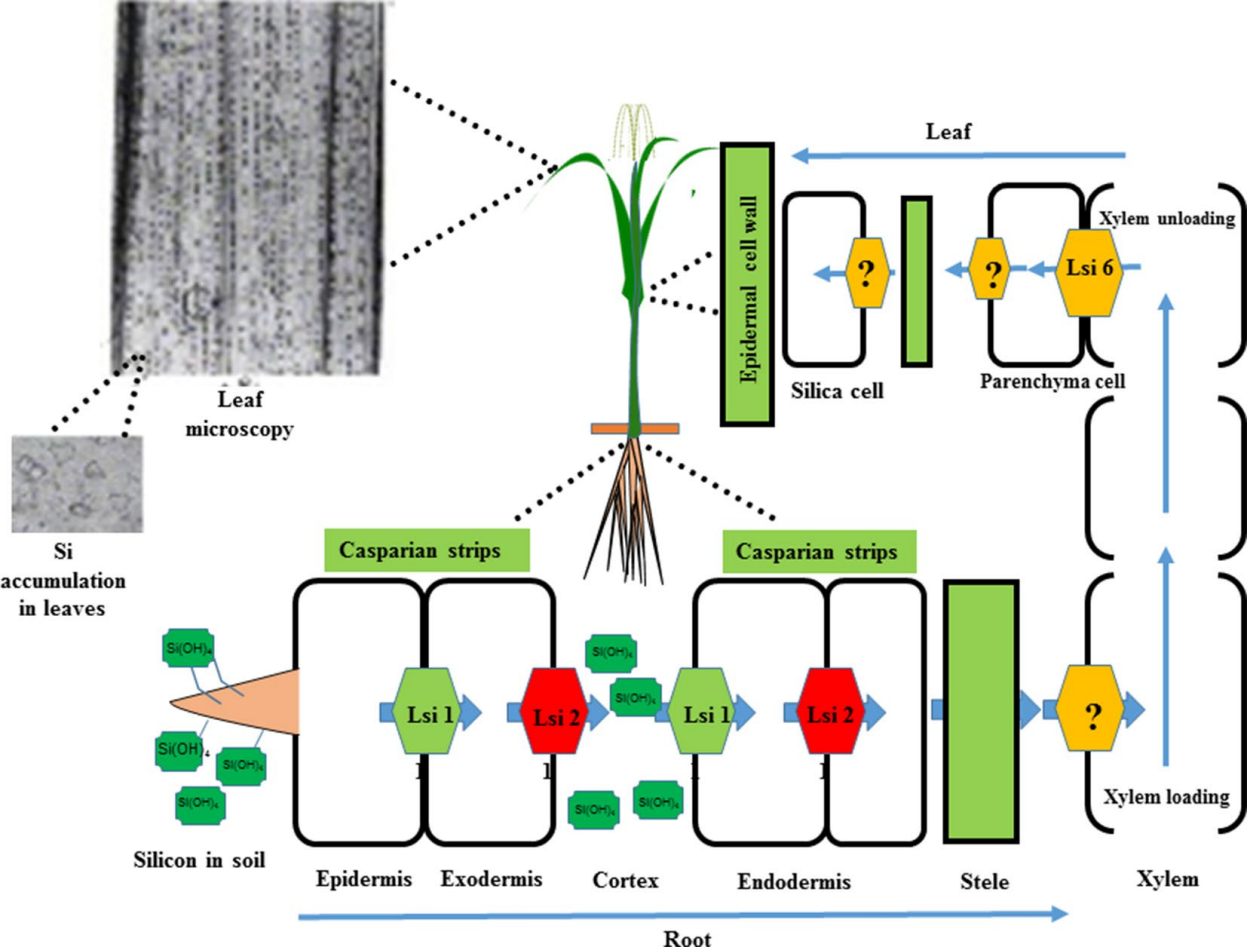
A schematic representation of Si uptake, transport, and accumulation in rice. Silicic acid from the soil solution is absorbed by the roots and transported to the root exodermis by the influx transporter (*Lsi1*) and subsequently released to the apoplast by the eﬄux transporter (*Lsi2*). Subsequently, it enters the root endodermis *via Lsi1* and is released to the stele portion of the root *via Lsi2*. Finally, silicic acid is translocated by an unknown transporter into the xylem and transported to the shoots *via* the transpiration stream. In the leaves, silicic acid is unloaded by another influx transporter (*Lsi6*) and localized in the xylem parenchyma cells of leaf sheaths and leaf blades. In the shoots and leaves, Si is transformed from an aqueous form (silicic acid) to solid amorphous silica (SiO2–nH2O) and primarily deposited in the cell walls of different tissues, such as leaf epidermal cells. Modified from Yan et al. (2018).

After absorption by the roots, Si is transported to other parts of the plant *via* the xylem of the roots (Ma and Yamaji, 2008). With the loss of water from a plant, inorganic amorphous oxides of silicic acid crystalize and precipitate, forming solid silica bodies called opal phytoliths that accumulate in the extracellular or intracellular spaces of plants, e.g. in the cell wall and trichomes (Ma et al., 2006; Cooke and Leishman, 2011). However, the mechanisms that prevent silicic acid polymerizing in the cell wall are not clear. Recently, it has been reported that the *low silicon* (*Lsi1*, *Lsi2*, and *Lsi6*) genes are responsible for Si uptake in the roots and its distribution to other organs in barley, rice, cucumber, and maize (Wang et al., 2015b). The *Lsi2* gene is expressed in the root endodermis and is considered a putative anion transporter (Ma et al., 2007; Mitani et al., 2011). On the other hand, the *Lsi1* and *Lsi6* transporters belong to the aquaporin family and have a major role in Si distribution in shoot and root tissue (Mitani et al., 2011). Furthermore, proton-driven transport activity has been reported in the *Lsi2* transporter (Ma et al., 2007), and it works as an Si/H^+^ antiport. The leaf epidermis and cell walls accumulate 90% of the total absorbed Si, which accounts for 10% of the dry weight of grass shoots (Yoshida, 1965; Ma and Takahashi, 2002; Raven, 2003). Silica that has accumulated intracellularly in the cytoplasm and vacuoles is stable even after plant decomposition and is abundant in soils (Lins et al., 2002). A schematic model of the Si transport system in rice is presented in **Figure 3**.

## Silicon-Mediated Mechanisms Involved in Increasing Salinity Tolerance in Crops

**Figure 4:**
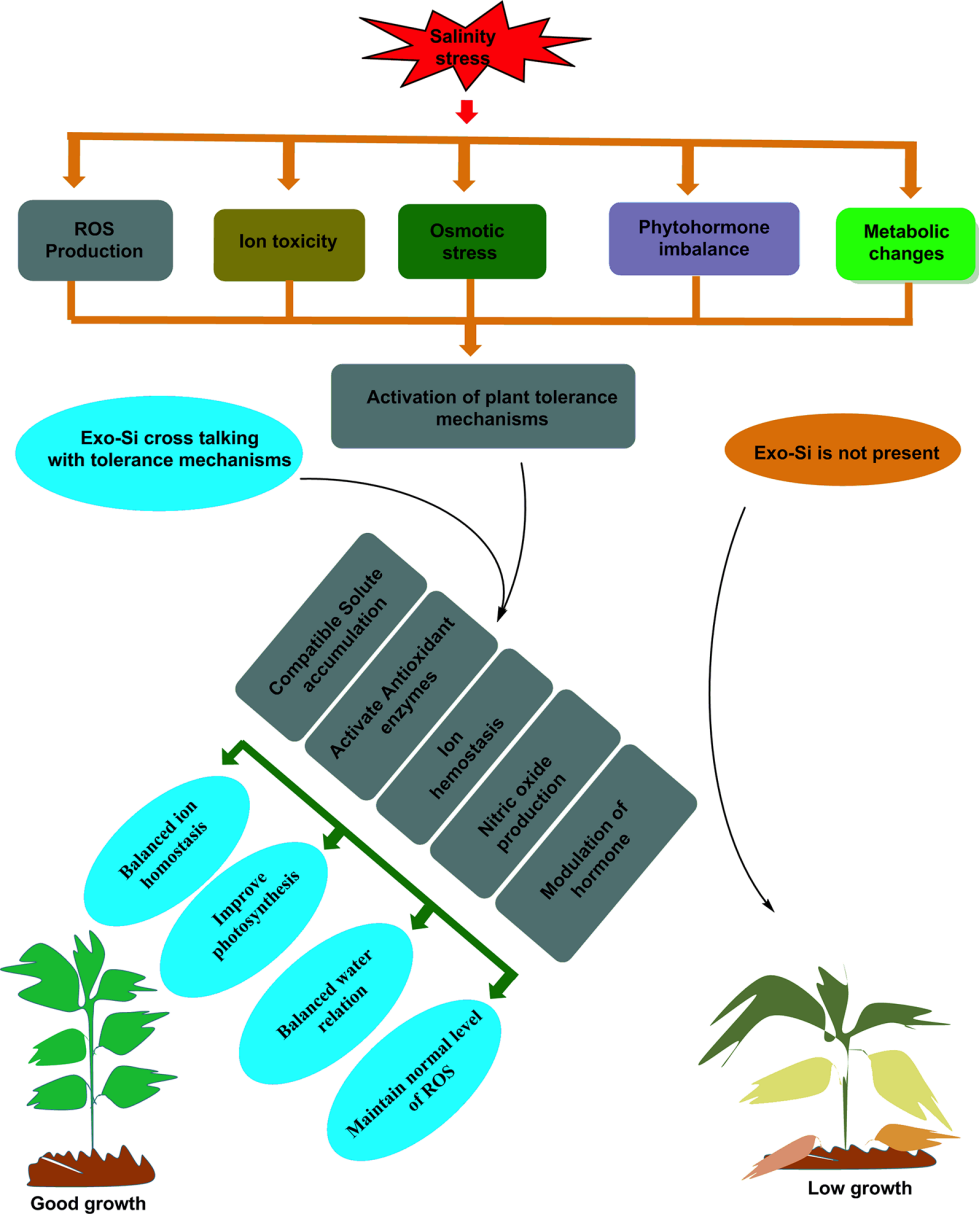
Schematic representation of silicon crosstalk with plants tolerance mechanisms during salinity stress.

Previous studies have reported the significant regulatory role of Si in numerous plant physiological processes under salinity stress (Rios et al., 2017). In fact, the many different complex biological functions reported by different studies suggests that the mechanisms by which Si improves the salt tolerance of plants have not been well studied. However, an improvement in the salt tolerance of different plant species following the exogenous application of Si has been reported, including in wheat (*T. riaestivum* L.; Tuna et al., 2008), barley (*Hordeum vulgare* L.; Liang et al., 2005a), maize (*Zea mays* L.), rice (*Oryza sativa* L.; Yeo et al., 1999), soybean (*Glycine max*; Lee et al., 2010), canola (*Brassica napus* L.; Haddad et al., 2018), spinach (*Spinacia oleracea* L.), cucumber (*Cucumis sativus* L.; Khoshgoftarmanesh et al., 2014), and tomato (*Lycopersicon esculentum* L.; Romero-Aranda et al., 2006), as summarized in **Table 2**. The exogenous application of Si improves plant growth either directly, i.e. by blocking the transport of Na^+^ ions into the plant, or indirectly, i.e. by activating different physiological processes to ameliorate the effect of salinity stress. The current understanding of the mechanisms underlying the Si-based mitigation of salinity-induced stress and its interaction with crops is shown in **Figure 4**.

**Table 2:** Effect of exogenous Si on plant stress tolerance mechanisms in various plant species under salinity stress.

Plant name	Source of silicon	Proposed Si-mediated tolerance mechanisms	Reference
***Triticumaestivum*** ** L.**	Potassium silicate	The results suggest that Si application hinders the uptake of Na^+^ and reduces the accumulation of proline, which could be due to the interaction of Si with Na^+^ uptake and proline accumulation. Hence, Si regulates the uptake of micro- and micronutrients under salinity stress.	Ibrahim et al. (2016)
***Lycopersicon esculentum***	Potassium silicate	The higher water levels in Si-treated plants could explain the higher plant growth and could be related to salt dilution within the plant and the consequent mitigation of salt toxicity effects.	Romero-Aranda et al. (2006)
***Cucumis sativus*** ** L.**	Sodium metasilicate	Supplementation of exogenous Si increases the accumulation of polyamines such as spermidine and spermine in cucumber plants. The enhanced polyamine accumulation with silicon application might play a role in modulating the antioxidant defense system and reducing oxidative stress, thus increasing the salt tolerance of cucumber plants.	Yin et al. (2019)
***Puccinellia*** ***distans***	Sodium metasilicate	The results suggest that Si application increases the levels of osmoregulatory organic solutes and reduces Na^+^ in sensitive tissue. Furthermore, Si improves plasma membrane activity *via* lower electrolyte leakage possibly through greater H^+^-ATPase activity, which could assist in Na^+^ secretion and exclusion from sensitive tissues. Si also increases the biosynthesis of lignin and cellulose levels, which could also facilitate Na^+^ secretion and exclusion.	Soleimannejad et al. (2019)
***Triticum aestivum*** ** L.**	Sodium metasilicate	In this study, the authors propose that improved growth in Si-treated plants can be attributed to reduced Na^+^ uptake, its restricted translocation to the shoots, and enhanced K^+^ uptake.	Tahir et al. (2011)
***Helianthus***	Sodium metasilicate	To alleviate the negative effects, silicon positively affects the uptake of nitrogen and antioxidant enzymes.	Conceição et al. (2019)
***Foeniculum vulgar mill.***	Sodium metasilicate	Silicon treatment improves the translocation of minerals, and the higher tolerance of salinity is believed to be associated with lower sodium concentrations and higher potassium concentrations.	Rahimi et al. (2012)
***Rosa hybrida***	Potassium silicate	Si increases tolerance by augmenting root hairs, which increase water uptake and consequently mitigates the osmotic imbalance. Si also hinders the uptake of Na^+^. In addition, Si boosts the antioxidant machinery, which could also be a reason for the increased tolerance in Si-treated plants.	Soundararajan et al. (2018)
***Triticum aestivum*** ** cv.**	Sodium metasilicate	The suppression effect of salinity stress was alleviated by exogenous Si by increasing the activity of antioxidant enzymes and by restoring the nutrient balance and osmotic potential.	Saleh et al. (2017)
***maize***	Metasilicic acid	The author suggests that silicon treatment improves growth mainly because of changes in ion accumulation, the enhancement of photosynthesis, and the regulation of antioxidant defense systems enzymes.	Khan et al. (2018)
***Cicer arietinum*** ** L.**	Potassium silicate	Exogenous application of Si hinders the uptake of Na^+^ and significantly improves the K^+^/Na^+^ ratio.	Garg and Bhandari (2016)
***Cucumis sativus*** ** L.**	Sodium silicate	Silicon improves transpiration rates and leaf water levels by maintaining the water balance. The study also suggests that silicon-mediated changes in root morphology may also account for the increased water uptake of silicon-treated plants.	Wang et al. (2015b)
***Solanum lycopersicum***	Metasilicic acid	Exogenous Si reduces the uptake of Na^+^ and Cl^-^ and boosts the antioxidant machinery in the roots of tomato, which facilitates root growth and hydraulic conductance, and thus improves the water status in the leaves.	Li et al. (2015)
***Wheat***	Calcium silicate	Si reduces the concentration of Na^+^ in wheat leaves. Hence, hindering Na^+^ uptake is a good indicator of salt tolerance in plants.	Ali et al. (2009)
***Glycine max*** ** L.**	Sodium metasilicate	Exogenous Si hinders the uptake of Na ions. Furthermore, the study demonstrates the interaction of Si with plant stress-related hormones. In this study, exogenous Si enhances the biosynthesis of ABA while reducing jasmonic acid biosynthesis. The regulation of these hormones under salinity stress is a possible reason for Si-based tolerance.	
***Glycine max*** ** L.**	Silicic acid	The results suggest that Si can increase the level of endogenous gibberellin and jasmonic acid while reducing salicylic acid. Hence, it is clear from this study that exogenous Si improves the tolerance of plants by regulating the biosynthesis of stress-related phytohormones.	Hamayun et al. (2010)
***Poa pratensis*** ** L.**	Sodium metasilicate	Silicon enhances leaf erection, which facilitates light penetration and promotes photosynthesis by significantly lowering the production of ethylene, which destroys chlorophyll and reduces plasma permeability.	Bae et al. (2012)
***Abelmoschus esculentus*** ** L.**	Silicic acid	Silicon confers salt tolerance on okra, possibly by enhancing the water status, improving antioxidant activity, and enhancing nitrogen metabolism.	Abbas et al. (2017)
***Triticum*** ***aestivum*** ** L.**	Calcium silicate.	The application of Si helps wheat plants to absorb high amounts of K^+^ and hinder the uptake of Na^+^ or its translocation.	Tahir et al. (2006)
***Oriza Sativa*** ** L.**	Sodium silicate	Silicon effectively reduces sodium ion transportation within the plant. It is also found that the reduction in silicon occurs not *via* transpiration but from reduced soil transport.	Yeo et al. (1999)
***Physalis peruviana*** ** L.**	Silicic acid	Silicon can act by increasing the capture of CO_2_ and maintaining the photosynthetic rate by increasing the stomatal density of the leaf. Silicon promotes the increase of this variable, indicating that it contributes to the reestablishment of stomata, reaching a number similar to the control.	Rezende et al. (2018)
***Acacia gerrardii Benth***	Potassium silicate	Silicon application improves the tolerance of *Acacia gerrardii* to salinity stress by improving the activity of both the enzymatic and non-enzymatic antioxidant defense systems. Si also reduces lipid peroxidation by enhancing the production of proline and glycine betaine.	Al-Huqail et al. (2017)
***Borago officinalis*** ** L.**	Sodium silicate	The addition of Si improves stress tolerance *via* various mechanisms such as improving the water status and efficiency of photosynthesis, increasing the production of proline while reducing that of glycine betaine, improving the antioxidant machinery, and reducing the uptake, transportation, and accumulation of sodium ions in sensitive tissue.	Torabi et al. (2015)
***Cucurbita pepo*** ** L.**	Potassium silicate	Exogenous Si application improves plant growth parameters by improving net photosynthesis by specifically hindering Na^+^ and Cl^-^ uptake and translocation to sensitive plant tissues, hence enhancing tolerance to salinity.	Savvas et al. (2009)
***Hordeum vulgare*** ** L.**	Potassium silicate	The presence of Si reduces the uptake of Na^+^ ions from the roots to shoots. Thus, Si-enhanced salt tolerance is associated with the selective uptake and transport of potassium and sodium by plants.	Zhu and Gong (2014)
***Ajuga multiflora***	Silicic acid	The addition of Si to the shoot induction medium significantly increases shoot induction. Thus, Si appears to promote shoot regeneration by altering the activity of antioxidant enzymes.	Sivanesan and Jeong (2014)
***Oryza sativa*** ** L.**	Sodium silicate	Exogenous Si improves tolerance by decreasing the sodium ion concentration in leaves.	Gong et al. (2006)
***Vicia faba*** ** cv.**	Sodium silicate	Si salt enhances stress tolerance by reducing Na^+^ translocation and decreasing transpiration under salinity.	Shahzad et al. (2013)
***Saccharum officinarum*** ** L.**	Calcium silicate	The results conclude that Si selectively interacts with Na^+^, and thus reduces Na^+^ uptake and translocation from the roots to shoots.	Ashraf et al. (2010a)

### Exogenous Si Regulation of the Antioxidant Machinery to Increase Salinity Stress Tolerance in Crops

The overproduction of ROS under salinity stress poses a threat to cells due to lipid peroxidation, protein oxidation, nucleic acid damage, enzyme inhibition, and the activation of programmed cell death pathways (Gill and Tuteja, 2010; Liang et al., 2018). To scavenge ROS, antioxidant enzymes such as CAT, SOD, and GPX, enzymes in ascorbate glutathione (AsA-GSH) cycles such as GR, MDHAR, APX, and DHAR, and non-enzymatic antioxidant molecules (ascorbate, alkaloids, flavonoids, phenolic compounds, proline, glutathione, α-tocopherol, and carotenoids) are activated (Kim et al., 2011; Wang et al., 2015b; Ahmad et al., 2019a). Several researchers have reported enhanced activity of the antioxidant machinery in plants to counteract oxidative stress induced by salinity (Zaefyzadeh et al., 2009; Chen et al., 2011). In a similar context, Si has been reported to alleviate the adverse effect of salinity by strengthening the antioxidant defense ability of crops.

The application of Si restores normal metabolism by reducing lipid peroxidation in maize, barley, and grapevine rootstocks (Liang et al., 2003; Moussa, 2006; Soylemezoglu et al., 2009; Kim et al., 2017). The reduction in lipid peroxidation under stress is thought to be the result of the maintenance of antioxidant enzyme production in plants. Hasanuzzaman et al. (2018) reported that the exogenous application of Si (1 mM) enhanced the activity of APX, MDHAR, GR, GST, DHAR, GPX, and CAT and raised AsA and GSH levels in *Brassica napus*. Similarly, higher production of SOD, CAT, and POD has been reported in *Abelmoschus esculentus* under salinity stress (Abbas et al., 2015). Recently, Ahmad et al. (2019a) described higher activity levels of SOD, CAT, APX, and GR in Si-treated *Vigna radiata* L. exposed to salinity stress. Gong et al. (2005) reported that Si application strengthens the antioxidant defense system and maintains normal physiological processes. Saqib et al. (2008) explained that Si enhanced the activity of antioxidative enzymes and reduced plasma membrane permeability.

To date, various studies have described enhanced antioxidant machinery in Si-treated plants under salinity stress. In *Glycyrrhiza uralensis*, the addition of Si increased POD and SOD activity and condensed MDA concentration (Li et al., 2016). However, the effect of Si on the antioxidant system is dependent on time, the concentration of Si, the severity of stress, and plant species. This can be illustrated by the results reported for two cultivars (‘Jinlu 4’ and ‘Jinyan 4’) of cucumber (*Cucumis sativus* L.). It was found that GPX and SOD activity decreased significantly in both cultivars under salt stress. In both varieties, Si did not affect GPX activity on the fifth day; however, activity increased significantly on the tenth day of treatment. Zhu et al. (2004) thus demonstrated that the activity of SOD, GPX, APX, DHAR, and GR increased with the application of Si under salt stress, but an increase in CAT activity was not observed. Liang et al. (2003) observed that CAT enzymatic activity increased in barley on day 2 under salt stress compared with the control regardless of whether Si was applied or not. On the day 4 and 6 of salt treatment, CAT activity reduced, but the addition of Si significantly rescued CAT activity. For grapevine plants under salt stress, the addition of Si did not affect SOD activity and reduced CAT activity, whereas APX activity was unchanged or increased depending on the cultivar (Soylemezoglu et al., 2009).

The studies suggest that Si supplementation can reduce the adverse effects of salinity by regulating the antioxidant defense system, which consequently decreases lipid peroxidation and ultimately maintains membrane integrity and decreases plasma membrane permeability. The literature suggests that Si-treated and non-Si treated plants exhibit different responses under salinity stress and that Si improves antioxidant activity, thus playing a protective role against salinity stress. Although important advances have been achieved in recent years, gaps still remain in the understanding of the interaction between exogenous Si and the plant antioxidant machinery. It should be noted that most of these results are from hydroponics experiments and require field trials. In addition to this, the effect of supplemented Si is known to be dependent on plant species, time, and organ, but the effect of Si on different isoforms is still not clear. Furthermore, most studies describe the effect of exogenous Si on antioxidant enzymes at a protein level. Hence, there is a need for comprehensive research to clearly demonstrate the interaction of exogenous Si with different isoforms of antioxidant enzymes at the protein and mRNA levels. If Si plays an active role in regulating ROS scavenging, it should be further specified when, where, and how this occurs (e.g. by regulating stress acclimation proteins and enzymes or by regulating the expression of the genes involved in managing ROS levels). Plants are affected not by a single stress factor but rather by a combination of harsh conditions. Elucidating the combination of salinity-induced changes in soil chemistry or in environmental contamination such as heavy metals is pivotal to understanding the interaction of exogenous Si with the antioxidant system. In the future, advanced imaging and ecophysiolomics techniques can lead to a better understanding of this interaction. Advanced approaches such as functional genomics, live-cell imaging, proteomics, and metabolomics will offer detailed insight into Si interactions with the antioxidant machinery.

### Silicon-Induced Reduction in Salinity Toxicity by Hindering the Uptake of Na^+^ From the Roots

Prolonged exposure to a high-salinity environment results in higher levels of Na^+^ and Cl^-^ and lower levels of other cations such as K^+^ and Ca^+2^, leading to a shift in the ion balance (Halperin and Lynch, 2003; Ahmad et al., 2019a). This ultimately results in changes to the K^+^/Na^+^ ratio in plants (Khan et al., 2000; Wang and Han, 2007). High concentrations of Na^+^ adversely affect plant metabolism and growth and lead to the overproduction of ROS (Mahajan and Tuteja, 2005). Recently, it has been reported that Si can ameliorate ion toxicity arising from salinity stress by enhancing K^+^ and reducing Na^+^ uptake (Tuna et al., 2008). It has been shown in several crops that the application of Si significantly reduces the accumulation of Na^+^ in the roots and hinders its translocation to sensitive plant tissues, consequently raising the K^+^/Na^+^ ratio.

**Figure 5:**
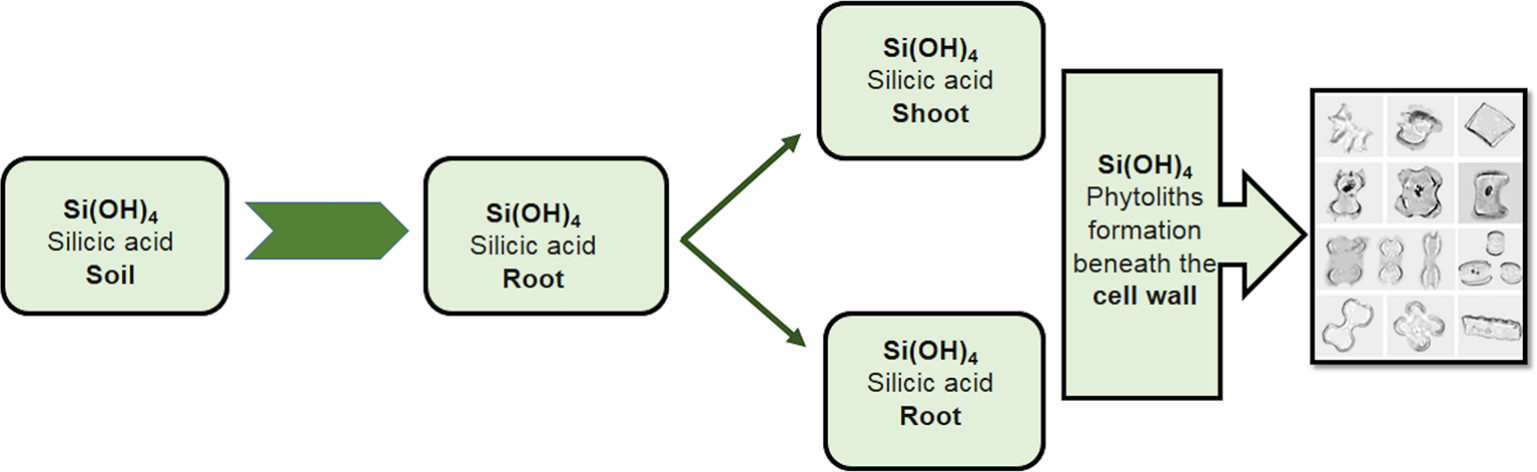
Accumulation of Si in different parts of plants. Silicon accumulates in the form of phytoliths or discrete silica bodies in different parts of a plant, e.g. the roots, leaves, and stems. This deposition takes place beneath the cell wall of the roots, where the discrete bodies of Si bind with the Na^+^, resulting in the increased uptake of K^+^ and the reduced transport of Na^+^ to the upper regions of the plants.

The regulation of the K^+^/Na^+^ ratio is a well-reported mechanism by which Si alleviates Na^+^ ion toxicity (Tuna et al., 2008). Silicon accumulates in the form of phytoliths or discrete silica bodies in different parts of the plant, e.g. the roots, leaves, and stem (**Figure 5**). This deposition takes place beneath the cell walls of the roots, where discrete Si bodies bind with Na^+^, resulting in the increased uptake of K^+^ and the reduced transport of Na^+^ to the upper regions of the plant. A study by Tahir et al. (2006) on the two wheat genotypes Auqab 2000 and SARC-5 found an increase in K^+^ ion concentrations and a decrease in Na^+^ ion concentrations following Si application under salt stress. Silicon has also been shown to play a role in Na^+^ ion detoxification by increasing the binding of Na^+^ to cell walls in both the salt-resistant wheat genotype SARC-1 and the salt-sensitive 7-Cerros (Saqib et al., 2008). Similarly, the exogenous application of Si decreases Na^+^ ion levels in alfalfa (*Medicago sativa* L.) roots but not in the shoots, though K^+^ levels notably increased in the shoots (Wang and Han, 2007). In rice, Gong et al. (2006) reported a dramatic reduction in Na^+^ concentrations in the shoots of salt-stressed plants following the application of Si. Gunes et al. (2007b) reported lower Na^+^ and Cl^-^ translocation from the roots to shoots in tomato plants following the application of Si. This mechanism has been reported by several studies, as shown in **Table 2**. Many nutrients have exhibited synergistic effects in wheat (Khan et al., 2015b) and facilitated its uptake within the plant body. Similarly, Si has demonstrated a synergistic effect with K^+^ by increasing its concentration within plant cells, such as in maize (Khan et al., 2015a) and wheat (Tahir et al., 2012).

The Na^+^/H^+^ antiporter is also known to play a vital role in maintaining low Na^+^ concentrations. This occurs with the removal of Na^+^ from the cytosol or its compartmentalization in vacuoles (Yue et al., 2012). Gene *SOS1* encodes plasma membrane Na^+^/H^+^ antiporters and has been cloned from *Arabidopsis* (Shi et al., 2000). The plasma membrane uses energy from ATP hydrolysis to pump H^+^ out of the cell, thereby generating an electrochemical H^+^ gradient, which is the main force driving Na^+^/H^+^ antiporter function. Tonoplast Na^+^/H^+^ antiporters play a role in Na^+^ compartmentation and are driven by *H* *^+^* *-ATPase* and *H* *^+^* *-PPase* in tonoplasts (Shi et al., 2000; Yamaguchi et al., 2013). Liang (1999) observed lower plasma membrane H^+^-ATPase activity in salt-stressed barley roots, but higher activity was observed following the application of Si to plants. Increased H^+^-ATPase levels facilitate Na^+^ export from the cell. Na^+^ compartmentation also plays an important role in preventing Na^+^ toxicity (Yamaguchi et al., 2013). Liang et al. (2005b) reported that the activity of H^+^-ATPase and H^+^-PPase in the tonoplast cells of barley roots was stimulated by the addition of Si under salt stress. This facilitates Na^+^ compartmentalization in vacuoles by tonoplast Na^+^/H^+^ antiporters. Mali and Aery (2008) reported that Si increases K^+^ uptake by increasing H^+^-ATPase activity in both hydroponics and soil. Recently, Soleimannejad et al. (2019) reported that Si improves plasma membrane activity by lowering electrolyte leakage, possibly *via* greater *H* *^+^* *-ATPase* activity, which could assist in Na+ secretion and exclusion from sensitive tissues. Therefore, under salt stress, the application of Si may lead to an increase in K^+^ levels and a decrease in Na^+^ levels in the cytoplasm due to H^+^-ATPase activity in the plasma membrane and tonoplasts and *H* *^+^* *-PPase* activity in tonoplasts. However, whether Si directly regulates the transport activity or expression of the Na^+^/H^+^ antiporter under salt stress remains unclear. In tomato, the addition of Si had no significant effect on Na^+^ and Cl^-^ concentrations in leaves, though it improved the storage of water in plants (Romero-Aranda et al., 2006). Tuna et al. (2008) reported that Si increased water content in wheat plants under saline conditions, whereas there was no effect on unstressed plants. The hydrophilic nature of Si to some extent may contribute to water restoration in salt-stressed plants.

These studies suggest that Si might alleviate the adverse effects of salinity by preventing Na^+^ uptake by the roots and subsequent movement to the shoots. The current literature suggests that the application of Si can provide protection against salinity stress and thus increase the yield and productivity of various crops. Silicon hinders Na^+^ translocation to aerial parts of a plant either by depositing Na^+^ in epidermal cells, creating a barrier to ion movement, or forming a complex between freely available Na^+^ and Si ions. However, some studies have linked the Si-mediated increase in tolerance to salinity with the activity of tonoplast H^+^-ATPase and H^+^-PPase in roots and plasma membrane H^+^-ATPase. The regulation of the K^+^/Na^+^ ratio by exogenous Si is thought to be a key mechanism in the Si-mediated increase in salt tolerance in crops. However, the effect of Si on Na^+^ dynamics across membranes and through extracellular spaces in plants needs to explored further. Many determinants have not been studied in sufficient detail in salt-stressed plants with or without the addition of Si, such as the Na^+^ signal perception process. Moreover, it is unclear whether the reduction in Na^+^ levels with the addition of Si is due to changes in the root structure and/or a reduction in the transpiration stream in the xylem, so this needs to be studied in more species. Moreover, the current literature lacks mRNA-level evidence for the role of Si in the regulation of the K^+^/Na^+^ ratio. More experiments are required to investigate the mechanisms involved in the regulation of this ratio in plants following the exogenous application of Si under saline conditions.

### Salinity Tolerance, Compatible Osmolytes, and the Role of Silicon

Of the widely accepted mechanisms for tolerance that have evolved to enable a plant to avoid the deleterious effects of stress, compatible solute accumulation marks a key position. Researchers have long sought to understand the role of compatible solutes that accumulate upon exposure of a plant to salinity stress, including proline (Kaur and Asthir, 2015), polyamines (PAs; Liu et al., 2015), carbohydrates (Negrao et al., 2017), glycine betaine (Hussain et al., 2018), and polyols (Parida and Das, 2005). These solutes are chemically diverse, uncharged in neutral pH, water-soluble, and accumulate in high concentrations during stress without inhibiting normal biochemical reactions (Zhang et al., 2004). They interact with membrane proteins or other protein complexes due to their hydrophilic nature. However, this interaction occurs without disturbing the normal structure and role of the protein (Bohnert and Shen, 1998). Compatible osmolytes are known to stabilize functional proteins, enzymes, protein complexes, and the membrane under salinity stress (Rajasheker et al., 2019). Osmotic adjustment has been shown to be an important component of stress tolerance, and the accumulation of osmoprotectants such as proline, glycine betaine, gamma-aminobutyric acid (GABA), and sugars has been regularly observed in different plant systems (Ashraf and Foolad, 2007; Chen and Jiang, 2010). The genetic engineering of metabolic conduits for a number of compatible solutes such as proline, glycine betaine, sorbitol, mannitol, and trehalose has led to the successful development of transgenic plants that exhibit increased resistance to drought stress, high salinity, and the cold (Bhatnagar-Mathur et al., 2008; Reguera et al., 2012). Interestingly, various studies have suggested that the application of exogenous Si can enhance the salinity stress tolerance of various crops by regulating the synthesis of compatible osmolytes (Seckin et al., 2009). Al-Huqail et al. (2017) reported that the application of Si protects Talh trees (*Acacia gerrardii* Benth) from the negative effects of high concentrations of salt by increasing the production of proline and glycine betanin, which help the plants to maintain their metabolic activity by conserving water levels in their tissues. However, several studies have shown that the levels of proline are lowered by the addition of Si in various species under salt stress, such as grapevine (Soylemezoglu et al., 2009), soybean (Lee et al., 2010), wheat (Tuna et al., 2008), barley (Gunes et al., 2007a), and sorghum (Yin et al., 2013). Lower levels of proline in salt-stressed plants following the addition of Si indicates the alleviation of stress damage. Yin et al. (2013) reported that the short-term application of Si significantly enhanced the levels of sucrose and fructose in sorghum plants under salt stress. Similarly, Si reversed the lower concentrations of the PAs putrescine and spermine in the roots of salt-stressed cucumbers (Wang et al., 2015b). Higher glycine accumulation following the application of Si illustrates its effect in modifying osmotic capacity and antioxidant levels in okra under saline conditions (Abbas et al., 2015). Recently, Yin et al. (2019) reported that the application of Si increases the accumulation of polyamines such as spermidine and spermine in cucumber plants. Their study suggested that enhanced polyamine accumulation might play a role in modulating the antioxidant defense system and reducing oxidative stress, thus increasing the salt tolerance of cucumber plants.

Silicon-mediated osmotic adjustment under salinity stress to protect subcellular structures has been considered a major mechanism underlying Si-based salinity stress tolerance; however, it is still debated whether higher osmolyte accumulation benefits crop yield (Seeraj and Sinclair, 2002). A variety of studies have reported conflicting results for the interaction between Si and compatible osmolytes such as proline. Some studies have concluded that the application of Si reduces the accumulation of compatible osmolytes such proline in different plants in the presence of salt, claiming that the lower synthesis of proline following the addition of Si reflects the alleviation of stress damage. On the other hand, other studies have found a higher accumulation of proline due to Si. Hence, more research is needed to clarify the relationship between the exogenous application of Si and the metabolism of compatible solutes and water transport.

### Silicon-Induced Improvement in Salinity Tolerance in Crops by Restoring the Rate of Photosynthesis

Photosynthesis is a fundamental process that takes place in the chloroplasts, resulting in the transformation of sunlight into energy to fuel a plant’s biochemical activities (Gong et al., 2010). The growth and productivity of plants largely depend on photosynthesis. From the large volume of data available on Si-induced improvement in shoot growth and net photosynthetic rate, it is reasonable to speculate that Si may maintain a high photosynthetic rate in salt-stressed plants. Previous reports have confirmed that salinity stress adversely disturbs the ultrastructure of chloroplasts, e.g. the dilation of thylakoid membranes and grana (Parida and Das, 2005), consequently disrupting the growth rate and productivity of plants.

Positive effects of Si on chlorophyll biosynthesis and photosynthetic machinery under abiotic stress have been widely reported. For example, under salinity stress, the exogenous application of Si has been found to improve photosynthesis in many species. Detmann et al. (2012) described the mechanisms behind the positive effect of Si on rice plants by analyzing photosynthetic gas exchange parameters alongside transcriptomic and metabolomic profiling. It was concluded that the rate of photosynthesis and the primary metabolism of a plant is enhanced by the application of Si. Silicon mitigates saline stress by maintaining stomatal conductance, transpiration, net photosynthesis, membrane permeability, and chlorophyll levels, which is partly due to the higher K^+^ ion concentrations and lower Na^+^ ion levels induced by the presence of Si in salt-stressed environments (Coskun et al., 2016). Tuna et al. (2008) reported that the application of Si to salt-stressed wheat restored chlorophyll levels. In barley, the application of Si increased chlorophyll levels and photosynthetic lead cell activity with or without salt stress (Nikolic et al., 2019). Advantageous effects of Si on the photosynthetic apparatus and pigments have also been observed in *Spartina densiflora* (Al-Aghabary et al., 2005; Mateos-Naranjo et al., 2013). Parveen and Ashraf (2010) studied various photosynthetic parameters, such as the net CO_2_ assimilation rate, stomatal conductance, the internal CO_2_ concentration in leaves, and the rate of transpiration in maize cultivars and reported that the exogenous application of Si improved all parameters under non-saline and saline regimes. Research on barley (*Hordeum vulgare* L.), rice (*Oryza sativa* L.), sugarcane (*Saccharum officinarum* L.), and wheat (*Triticum aestivum* L.) crops has shown that Si deposited in leaves is able to improve the potential and efficiency of photosynthesis by opening the angle of the leaves, decreasing self-shading, and keeping the leaf erect, thus it plays an important role in increasing the growth and yield of crops (Soratto et al., 2012).

The application of Si also improves plant photosynthetic machinery under salinity stress either by lowering ion toxicity and ROS accumulation to maintain the structure and function of the organelles that are responsible for photosynthesis or by increasing stomatal conductance, the transpiration rate, and the number and size of the stomata. In addition, Zhu et al. reported that the application of Si reduces starch and soluble sugar levels in cucumber leaves while increasing starch levels in the roots. This is because salinity stress increases the accumulation of photosynthetic products such as sucrose and starch in the leaves by affecting their transport and allocation, causing feedback inhibition of the photosynthesis process. However, the available literature lacks strong evidence for the role of Si in the synthesis, translocation, and allocation of photosynthetic products. Thus, advance molecular biology, proteomics, and advanced imaging techniques should be employed to further explore the mechanisms by which Si affects carbohydrate metabolism.

In conclusion, Si modifies the gas exchange process, decreases Na^+^ accumulation, enhances chlorophyll levels, scavenges ROS, and regulates carbohydrate metabolism, all of which ultimately enhances the photosynthesis of salt-stressed plants. However, this improvement depends on the plant species, salt-stress levels, and the application levels of the Si. Further studies are required to understand the role of exogenous Si in carbohydrate metabolism and its positive effect on photosynthesis under salinity stress. In addition, in-depth research is required to collect strong evidence for the involvement of Si in the improvement of the photosynthetic machinery under both salinity stress and combined stress, such as salinity in conjunction with heavy metals, drought, or heat.

### Silicon and the Regulation of Endogenous Phytohormones Under Salinity Stress

The impact of silicon on endogenous phytohormones in response to stress conditions has been widely reported. The effect of Si on endogenous phytohormones such as GA, ABA, JA, ET, SA, BR, and IAA has commonly been studied in the context of the response to stress situations (Fahad et al., 2015). Although the protective role of these hormones has been studied extensively for a variety of stress types, the crosstalk between Si and phytohormones under salinity stress is poorly understood. However, studies have reported that the application of Si might enhance stress resistance by modifying phytohormone homeostasis (Van Bockhaven et al., 2012).

ABA is a stress hormone that affects gene expression (Parida and Das, 2005) in response to salt stress (Wang et al., 2001b; Dodd and Davies, 2010). The short-term application of Si downregulated JA and upregulated ABA after 6 and 12 h in rice plants under stress. The application of Si in combination with salt stress transiently increased the expression of the ABA biosynthesis-related genes *zeaxanthin epoxidase* and *9*-*cis*-*epoxicarotenoid oxygenase 1* and *4* (*ZEP*, *NCED1*, and *NCED4*) compared to salt stress alone in rice (Kim et al., 2014b). The findings of the study conducted by Kim et al. (2011) on *Oryza sativa* suggest that the exogenous application of Si can modulate salinity-induced stress by regulating the phytohormonal response of plants, e.g. the upregulation of ABA (Maillard et al., 2018), with the effects dependent on time. However, the link between salt tolerance and Si-mediated changes in plant hormones has yet to be investigated. Lee et al. (2010) reported that ABA levels increase in soybean plants under salt stress but decrease when Si is applied. Furthermore, it was concluded that GA levels decrease under salt stress but increase with the application of Si. In soybean plants, Si alleviates the negative effects of NaCl on the growth of plants by enhancing endogenous GA_3_ and lowering ABA levels (Lee et al., 2010). Adverse NaCl effects are reduced significantly with the application of Si by increasing bioactive gibberellin (GA_1_ and GA_4_) levels, but the levels of JA, which increase under salinity stress, decline sharply when plants are supplemented with Si (Zhang et al., 2018). Another report demonstrated that JA and SA concentrations decrease and increase, respectively, in Si-treated soybean plants under salt stress (Kim et al., 2014a). Thus, the regulatory effect of Si on salt tolerance levels in crop plants *via* the regulation of endogenous phytohormone signaling has been proposed. However, further research is required to clarify the relationship between Si, stress tolerance, and phytohormonal signaling, particularly with SA, JA, ETHY, BR, and melatonin.

## Conclusion and Future Prospects

Saline environments have adverse effects on plant growth and yields worldwide. Plants respond to high-salinity stress using various mechanisms, including the regulation of Na^+^ uptake and translocation, the activation of their antioxidant defense system, compatible solute accumulation, osmotic regulation, the regulation of phytohormone synthesis, and the induction of various stress-signaling cascades. All of these responses play an important role in plant adaptation to salt stress. Silicon has been proven to increase tolerance to salinity stress by regulating various biochemical and physiological processes, such as the Na^+^ balance, water status, reactive oxygen species, photosynthesis, phytohormone levels, and compatible solutes in plants.

Various studies have shown that Si supplementation benefits the development of different plant species, specifically when they are exposed to ecological stresses. Of the various Si-mediated salinity stress tolerance mechanisms, the available literature suggests that the application of exogenous Si (i.e. foliar and root application) improves salinity tolerance in plants either by enhancing the activity of antioxidant enzymes or blocking Na^+^ uptake and translocation. Furthermore, we conclude that the positive effect of exogenous Si depends on a plant’s stress tolerance levels, which vary between species. This might be due to the differences in Si uptake capabilities among different species. Despite this, the effect of Si on plant stress tolerance generally depends on Si concentration, stress intensity and duration, Si application methods, and the cultivation methods used for experimental materials (e.g. soil culture or hydroponics).

It is known that approximately 20% of irrigated land is salt-affected which is one-third of all food-producing land. It has been estimated that about half of all fertile land will be affected by salinity by the middle of the 21^st^ century. To overcome salinity stress in the future, Si-mediated salt tolerance mechanisms will help to enhance salt stress tolerance in various crop plants. However, many determinants and regulatory mechanisms have not been studied in detail and thus need further elucidation. This paper suggests the following future research recommendations and prospects for Si-mediated salt tolerance in plants:

1. With the development of advanced omics technologies, more detailed research is required to explore Si-mediated salt tolerance at the transcriptome, proteome, and metabolome levels. 
2. *SOS* pathways have a vital role in salinity stress tolerance. However, the interaction of exogenous Si with plant SOS signaling pathways and other salt stress sensors remains obscure.
3. Most previous research has studied the role of Si in salt stress on its own and in the short-term. However, in nature, plants are exposed to multiple stresses simultaneously. Establishing stress tolerance over a longer period of time would be ideal for predicting and reacting to changing global climatic conditions, especially where one form of stress leads to another. Thus, the role of Si in long-term plant responses under multiple stresses requires in-depth research.
4. More work is needed to analyze the regulatory mechanisms of Si in salt-induced osmotic stress. Efforts should be made to clearly demonstrate how Si regulates osmotic adjustment under salinity stress. The genetic engineering of metabolic conduits for a number of compatible solutes, such as proline, glycine betaine, and sorbitol, could also be used to produce salt-tolerant plants.

In addition, Si-associated molecular and transcriptional changes at the plant level are yet to be elucidated, including the various metabolomic and proteomic changes in different plant organs. Currently, the mechanisms underlying the Si-mediated alleviation of salt stress in plants is poorly understood at the molecular and genetic levels. In addition, more focus is needed on the effects of Si under field conditions rather than greenhouse or laboratory studies.

## Author Contributions

AK and ALK wrote the manuscript. SM and AA-H edited the manuscript. Y-HK organized graphical presentation. AA-R arranged funding.

## Funding

The author(s) acknowledge the financial support of the Research Council Oman for research grant to corresponding authors.

## Conflict of Interest

The authors declare that the research was conducted in the absence of any commercial or financial relationships that could be construed as a potential conflict of interest.
